# Clinical Application Evaluation of Elecsys^®^ HIV Duo Assay in Southwest China

**DOI:** 10.3389/fcimb.2022.877643

**Published:** 2022-05-18

**Authors:** Mei Yang, Wenjuan Yang, Wu Shi, Chuanmin Tao

**Affiliations:** Department of Laboratory Medicine, West China Hospital, Sichuan University, Chengdu, China

**Keywords:** diagnostic performance, southwest China, HIV duo assay, human immunodeficiency virus, cutoff index

## Abstract

**Background:**

HIV/AIDS continues to be a serious health concern of morbidity and mortality globally, and novel HIV testing is still an important component of diagnosing HIV earlier and reducing the spread of HIV. The Elecsys^®^ HIV Duo assay is a 4th generation assay that can detect both HIV-1 p24 antigen (Ag) and HIV-1/2 antibody (Ab) in parallel and show the subresults for the Ab and Ab units.

**Objectives:**

To evaluate the clinical performance of the Elecsys^®^ HIV Duo assay on the new cobas E 801 analyzer using a large number of clinical samples from a population in southwest China.

**Methods:**

We collected testing results and information from all patients in a large general hospital. All eligible clinical specimens were first analyzed using the Elecsys^®^ HIV Duo assay. The test results are given either as reactive or nonreactive as well as in the form of a cutoff index (COI). All initially reactive specimens were retested in duplicate with a 3rd-generation kit. Supplementary tests were divided into Ab confirmation tests and HIV-1 nucleic acid tests. GraphPad Prism and Python were used for plotting, and SPSS 21.0 software was used for statistical analysis.

**Results:**

A total of 186391 specimens were received, and 436 patients were confirmed to be positive for HIV. Among the 86 cases with contact history available, there were more males than females, and heterosexual transmission was the most common route of HIV infection. The Elecsys^®^ HIV Duo assay displayed 99.94%, 99.93% and 99.98% specificity for inpatient, outpatient and physical examination patients, respectively. The median COI ratios of the false-positive group were significantly lower than those of the true-positive group.

**Conclusions:**

The Elecsys^®^ HIV Duo test (Cobase801 analyzer) differentiates the detection of HIV-1 p24 Ag and HIV-1/2 Ab with high specificity and facilitates the diagnosis of patients with early HIV infection. Therefore, the Elecsys^®^HIV Duo test is used for differentiation of antigen and antibody reactivity, making it suitable for routine clinical diagnosis.

## Introduction

HIV/AIDS continues to be a serious health concern of morbidity and mortality globally. Since 2010, the number of new HIV infections has fallen by 31%, from 2.1 million to 1.5 million in 2020. As a result, according to global data, much progress has been made in the prevention and treatment of AIDS. Morbidity, mortality and new HIV infections are declining year by year globally. Although the rate of HIV infection has decreased significantly compared with the early years, the current situation is still not optimistic. According to the World Health Organization, 37.7 million people were infected with HIV in 2020 worldwide; the number of deaths from AIDS topped the list of notifiable infectious diseases in China (Xu et al., 2020). A total of 1,045 million living HIV/AIDS patients were reported in China by October 31, 2020. The Joint United Nations Program on HIV/AIDS (UNAIDS) has launched the 90-90-90 targets, the first of which is that 90% of people living with HIV (PLHIV) know their infectious status and are the most challenging. However, as of 2019, the reality was that only approximately 81% of patients were aware of their HIV status (Lu et al., 2020). Additionally, an effective strategy for HIV testing forms risk assessment of transmission of blood-borne pathogens for hospital staff who are exposed to blood and body fluids (WHO Guidelines Approved by the Guidelines Review Committee, 2010). Thus, universal screening for HIV-infected individuals (both known and unknown), who may or may not be aware of their infection status, is recommended so that patients need to be linked to care, retained in care, take antiretroviral medications, adhere to the prescribed regimens, and receive prophylaxis against opportunistic infections (Chang et al., 2013).

The HIV-1 Ag is the core protein of HIV-1. It is a 24-25 kDa protein encoded by the gag gene, which plays an important role in the packaging and maturation of the virus. Similar to RNA, it can be detected before seroconversion, so it becomes a marker of early HIV infection (Rafferty et al., 2019). HIV-1 Ag typically appears around the first 2 weeks post-infection (Fiebig et al., 2003; Cohen et al., 2010), and the concentration reaches the peak in one to two months later. As the earliest detected immune marker in serum, accurate detection of HIV-1 p24 Ag is conducive to the early diagnosis of HIV and can effectively shorten the window period. Serum HIV antibodies (Ab) include IgM and IgG antibodies, which are appeared approximately 3 and 6 weeks post-HIV infection, respectively (Alexander, 2016). As HIV Ab reactions usually persist over the course of infection, the initial screening and diagnosis of HIV mainly rely on different methods to detect HIV Ab in China (Eshleman et al., 2019).

The CDC recommends a complex HIV detection algorithm. The basic principle of this algorithm is to perform preliminary screening based on Ag/Ab. Based on the screening results, if the initial test results are reactive, the differentiation analysis of HIV-1/2 Ab and nucleic acid testing (NAT) is performed. However, if the screening results are nonreactive, no additional testing is required, and the result is considered negative. Therefore, a highly sensitive test is a key factor in eliminating false negative results.

Newly approved 4th-generation HIV tests have entered the international market and are commercially available, and they are commonly used in many laboratories to screen for HIV infection based on new diagnostic algorithms (Miedouge et al., 2011; Liu et al., 2016; Chacón et al., 2017). 4th-generation HIV assays can detect different HIV type 1 (HIV-1) non-B subtypes (group O Abs), HIV-1 p24 Ag, HIV-1/2 IgM, and IgG Ab simultaneously with extremely high sensitivity and specificity (Alexander, 2016; Stone et al., 2018). Therefore, compared to other methods (Cohen et al., 2010), 4th-generation HIV assays decrease the “window period” to 11–14 days post-exposure and enable the testing of acute and early HIV infection (Alexander, 2016; Stone et al., 2018). However, some 4th-generation Ag and Ab combo assays only provide a single result and cannot distinguish between HIV Ag and Ab readings and therefore have interpretation difficulty (Alexander, 2016).

The Elecsys^®^ HIV Duo assay (Roche Diagnostics) for use on the Cobas E 801 analyzer (Roche Diagnostics, Penzberg, Germany) is a new 4th-generation assay that can detect both HIV-1 p24 Ag and HIV-1/2 Ab of clinical patients samples in parallel by two different reactions with a rapid test time of 18 min. This new instrument allowing for continuous loading of reagents and consumables can not only quickly confirm whether a patient is in an acute stage but also facilitate doctor–patient communication and early treatment. Specifically, this instrument has high normal running time and requires less manual time. A previous study (Muhlbacher et al., 2019) suggested that the Elecsys^®^ HIV Duo assay is highly sensitive for the early detection of HIV, which was assessed at five laboratories from four different countries and compared with other available 4th-generation assays. Another study (Zhang et al., 2020) also showed that the Elecsys^®^ HIV Duo assay had excellent performance for 3039 serum samples from Chinese patients.

Our study is unique in that this is the first retrospective study to evaluate the clinical performance of the Elecsys^®^ HIV Duo assay on the new cobas E 801 analyzer using a large number of clinical patients samples from a population in southwest China.

## Methods and Materials

### Elecsys^®^ HIV Duo

Elecsys^®^ HIV Duo is an enzyme-linked assay using a sandwich principle that allows simultaneous detection of HIV-1 p24 Ag as well as HIV-1 (including group O) and HIV-2 Ab. Biotinylated and ruthenium-labeled anti-p24 monoclonal antibodies were used for the detection of p24 Ag. Biotinylated and ruthenium-labeled HIV-specific proteins and D-peptide-bound streptavidin are used for HIV Ab detection. The main results of Elecsys^®^ HIV Duo are automatically calculated using the e-flow system unique to the Cobas E 801 platform. HIV Ag and antibodies can be used as an aid in the selection of the confirmation algorithm for reactive samples. The results were automatically calculated by Elecsys^®^ software. COI values (sample signal value/critical value) by comparing the electrochemiluminescence signal of the sample with the critical value obtained in calibration. Elecsys^®^ software automatically calculates HIV DUO primary results based on secondary results. Calculation formula: $\text{HIV }\mathrm{DUO}[\mathrm{COI}]=\mathrm{HIV}\;\mathrm{DUO}[\mathrm{COI}]=\sqrt{{\left(\text{HIV Ag}[\text{COI}]\right)}^{2}+{\left(\mathrm{HIV}\text{ Ab}[\text{COI}]\right)}^{2}}$.

### Study Design

This retrospective study was conducted in a large general hospital with a catchment population of approximately 16.33 million inhabitants in Sichuan, China. The hospital’s laboratory has the HIV confirmation laboratory in China, which was certified as a laboratory by the College of American Pathologists (CAP) in 2006, and has also passed the competency verification of the Clinical Laboratory Center of the National Health Commission. We collected testing results and data from all patients who underwent initial screening for HIV discriminant Ag and Ab and HIV complementary testing (Ab confirmatory test or HIV-1 nucleic acid test) at this hospital between January 2021 and October 2021. In addition, by searching the electronic medical record management system of the hospital, we collected the patient’s medical records, such as the reason for seeing a doctor, medical information, clinical symptoms, past medical history and disease course records.

The study was conducted in full compliance with the principles of the Helsinki Declaration and local regulations. The study protocol was approved by ethics committee of the West China Hospital of Sichuan University. Exemption for obtaining informed consents from subjects was granted as a retrospective analysis of routinely collected programmatic data, and there was no direct contact with patients and not interfere with the clinician’s diagnosis and treatment.

### Study Population and Specimens

HIV screening subjects: A total of 186,391 patients were inpatients, outpatients, emergency patients, and healthy physical examination participants who received an initial screening test for HIV differentiated Ag and Ab in the hospital from January 2021 to October 2021.

HIV confirmed subjects: A total of 578 patients tested reactive for HIV differentiated Ag and Ab during the initial screening period from January 2021 to October 2021.

All collected fresh clinical specimens were centrifuged at 3500 rpm/min for 10 min, and then serum or plasma was separated for detection. They are stored in the refrigerator at 4-8°C for up to 1 week and at -80°C for longer periods prior to testing.

### Screening Procedures

**Figure 1:**
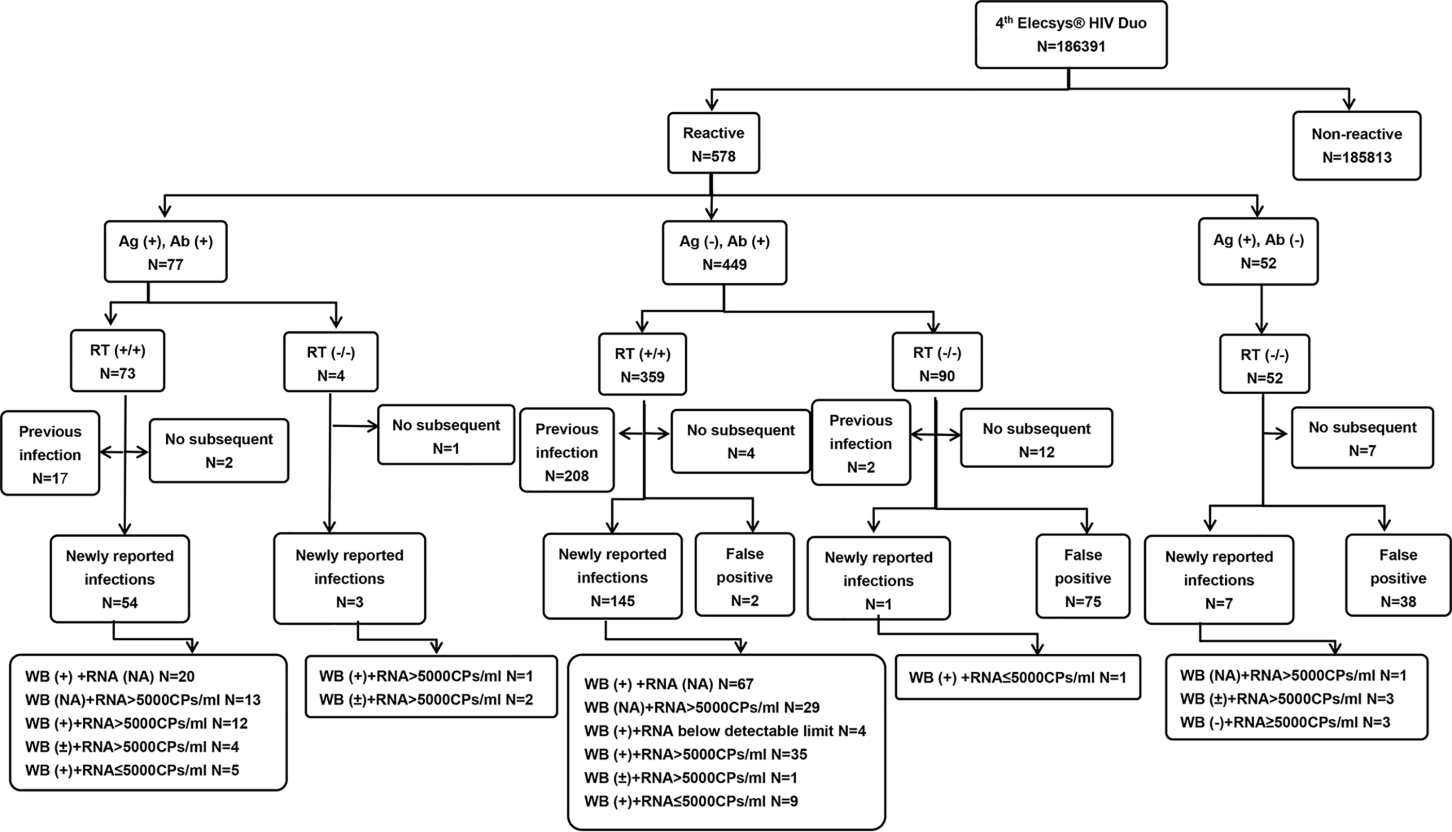
The distributions of the results of the Elecsys^®^ HIV Duo assay.

All eligible clinical specimens were first analyzed using the Elecsys^®^ HIV Duo assay, performed on the Cobas E 801 platform (Roche Diagnostics, Mannheim, Germany), following the manufacturer’s instructions. The screening procedure and detection algorithm are shown in **Figure 1**. The test results were given either as reactive (COI≥1.0) or nonreactive (COI<1.0) as well as in the form of a cutoff index (COI). All initially reactive specimens were retested in duplicate with a 3rd-generation kit-colloidal gold method—anti-HIV (Livzon Diagnostics Inc.) according to technical specifications for national AIDS testing published by Chinese Center for Disease Control and Prevention. Specimens were considered repeatedly reactive [quality control line (C) and test line (T) appear simultaneously) and nonreactive (only one quality control line (C) does not appear detection line (T)].

### Supplementary Test

Supplementary tests are divided into Ab confirmation tests and HIV-1 nucleic acid tests. The HIV-1 nucleic acid test includes qualitative and quantitative tests. Recommended confirmatory algorithms need to be confirmed for test results. Western Blot HIV blot 2.2 (MP Diagnostics, Singapore) was used as the HIV confirmatory test, which can detect IgG antibodies specific to viral Ag. On the basis of the manufacturer’s specification, the results of WB were reported as HIV-1 positive [the presence of at least two bands, including two env bands (gpl60/gp41 and gpl20) or two gag bands (p17, p24, p55) or two pol bands (p31, p51, p66)]; HIV-1/2 positive (the presence of HIV-1 positive bands and clearly visible HIV-2 specific bands); negative (the absence of any of the specific bands or only p17 antibodies were detected); indeterminate (reactivity to any of the bands but not meeting the criteria for a positive indeterminate result); inconclusive but suggestive of HIV-2 positive infection (any specific band is present, but not sufficient to be positive, and the HIV-2-specific band is clearly visible).

When NAT is used as supplementary test: suspected HIV-infected patients who respond to the screening test but have uncertain or negative Ab confirmatory result should be judged based on NAT. A total of 123 patients were voluntarily tested for HIV-1 RNA.

### Statistical Analysis

GraphPad Prism and Python were used for plotting, and SPSS 21.0 (SPSS Inc., Chicago, IL) software was used for statistical analysis. Quantitative data were confirmed and expressed as the median and interquartile range (IQR). Comparisons between continuous variables were made using the t test, depending on the normality of the distribution. A nonparametric test was used to analyze those that did not conform to a normal distribution. The chi-square test or Fisher’s exact probability method was used for counting data. The test level is set as α=0.05. HIV screening positive rate = the number of HIV initial screening positive cases/the number of all tested cases; HIV confirmed positive rate = number of HIV confirmed positive cases/number of all tested cases; positive rate of HIV Ab retest = number of Ab retest positive cases/number of all tested cases; incidence of HIV uncertainty = number of HIV uncertainty/number of all tested cases; efficiency of HIV Ab retest = number of false positive cases of negative Ab retest/(number of positive cases screened - number of positive cases of Ab retest): this index can reflect the ability of retest to exclude false positive samples from screened positive samples; confirmation efficiency of HIV testing process = (number of HIV-positive confirmed cases + number of HIV-negative confirmed cases)/number of retested positive cases: this index reflects the ability of subsequent tests (WB or HIV-1 RNA detection) to remove false positives from retested positive samples after retesting; Positive predictive value = true positive number/(true positive + false positive number).

## Results

### The Testing Algorithm and Distributions of Specimens Tested in the Elecsys^®^ HIV Duo Assay

As shown in **Figure 1**, a total of 186,391 specimens were received from January 1 to October 31. Of the 186,391 specimens tested, 185,813 (99.69%) were nonreactive. A total of 578 (0.31%) specimens were repeatedly reactive; Ag (+) and Ab (+), Ag (-) and Ab (+), Ag (+) and Ab (-) specimens were 77, 449, 52 cases, respectively. After excluding 26 samples that were lost to follow-up, 552 cases were confirmed to be reactive for screening. Then, the colloidal gold rapid detection method was used for subsequent repeated detection. After that, the patients were divided into previous HIV infection, newly reported HIV infection, false positive and no follow-up groups by means of case information query and telephone communication. In 210 (36.33%) patients with newly reported HIV infection, two complementary tests, WB and HIV RNA, were used for final determination. Finally, 436 patients were confirmed to be positive for HIV, and 116 cases were negative for supplementary tests.

### Detailed Results for 186391 Samples Assayed and the Overall Specificity of Each Assay Calculated by Combining the Data

**Table 1** showed HIV screening, HIV prevalence and performance evaluation of the Elecsys^®^ HIV Duo method. Compared with inpatients and physical examination patients, outpatients had the largest number of HIV-positive screening patients, accounting for 0.46% of the positive screening patients. Similarly, the positive rate of HIV confirmed by supplementary testing in outpatients was the highest, reaching 0.37%. The overall specificity of the Elecsys^®^ HIV Duo assay across all 186391 samples of inpatients, outpatients and physical examination patients in this study relative to the final HIV status was 99.94%, 99.93% and 99.98%, respectively.

**Table 1:** Detailed results for 186391 samples assayed and the overall specificity of each assay calculated by combining the data.

	Inpatient	Outpatient	Physical examination	Total
Screening non-reactive (n)	97864	68559	19390	185813
Screening reactive (n)	241	314	23	578
Screening reactive rate (%)	0.25	0.46	0.12	0.31
Total screening (n)	98105	68873	19413	186391
Confirmed negative (n)				
Confirmed positive (n)	174	252	10	436
Confirmed positive rate (%)	0.18	0.37	0.05	0.23
Total Confirmed (n)				
Specificity (%)	99.94	99.93	99.98	99.94

### Median (Quartile Range) Cutoff Index (COI) Ratios of All 522 WB or Subsequent Supplement Detections Confirmed Individuals

**Table 2** showed that the differences in median COI ratios between true-positive and true-negative results of HIV Duo and HIV-1/2 Ab were large. As mentioned above, HIV infection status was determined by WB, follow-up, HIV-1 RNA, or HIV-1 p24 Ag. As seen in **Table 2**, the median COI value in true-positive group of HIV Duo, p24 Ag, and HIV-1/2 Ab were 522.00 (IQR:154.50,1397.50), 0.19 (IQR:0.16,0.31) and 522.00 (IQR:152.75,1388.75), respectively., while value in false-positive group was 2.02 (IQR:1.44,4.10), 0.19 (IQR:0.17,1.76) and 1.31 (IQR:0.10,2.03). Notably, there were significant differences in terms of true positive and false positive for HIV Duo (P=0.000) and HIV-1/2 Ab (P=0.000).

**Table 2:** Median (quartile range) of cutoff index (COI) ratios of all 522 WB or subsequent supplement detections confirmed individuals.

Median (quartile range) of COI ratios	HIV Duo	P24 Ag	Ab	Total (n)
True positive	522.00 (154.50,1397.50)	0.19 (0.16,0.31)	522.00 (152.75,1388.75)	436
False positive	2.02 (1.44,4.10)	0.19 (0.17,1.76)	1.31 (0.10,2.03)	116
*P*	0.000	0.247	0.000	–

### Predictive Value of the Stratified COI Value for HIV Infection

As shown in **Table 3**, for HIV Duo, the COI values of 43.30% of all tested specimens were less than 200. When the COI ranged from 1 to 4.99, 97.85% of the specimens were negative at the initial screening, while when the COI was between 5 and 14.99, 70% of the specimens were negative. In addition, the COI values of negative specimens were mostly in the range of 1-49.99, and only 2 specimens had COI values ≥50.

**Table 3:** Results of all 552 WB or subsequent supplementary test-confirmed individuals in relation to cutoff index (COI) ratios.

COI ratios	Positive n (%)			Negative n (%)		
	HIV DUO	HIV-1/2 Ab	HIV1-P24 Ag	HIV DUO	HIV-1/2 Ab	HIV1-P24 Ag
0-0.99	–	7 (15.22)	356 (82.22)	–	39 (84.78)	77 (17.78)
1-4.99	2 (2.11)	2 (2.94)	34 (55.74)	93 (97.89)	66 (97.06)	27 (44.26)
5-14.99	6 (30)	8 (42.11)	21 (80.77)	14 (70)	11 (57.89)	5 (19.23)
15-49.99	36 (83.72)	32 (100.00)	16 (76.19)	7 (16.28)	0	5 (23.81)
50-99.99	43 (97.73)	39 (100.00)	6 (85.71)	1 (2.27)	0	1 (14.29)
100-199.99	36 (97.30)	36 (100.00)	3 (75.00)	1 (2.70)	0	1 (25.00)
200-399.99	67 (100)	66 (100.00)	–	0	0	–
>399.99	246 (100%)	246 (100.00%)	–	0	0	–
Total n (%)	436 (78.99)	436 (100.00)	436 (78.99)	116 (21.01)	116 (21.01)	116 (21.01)

Ab, antibody; Ag, antigen.

For HIV-1/2 Ab, 43.48% of all tested specimens were less than 200. When the COI value was between 1 and 4.99, 97.06% of the specimens were negative in the initial screening, while the COI value was between 5 and 14.99, and the rate of negative specimens and true positive specimens was equal. Specifically, only the specimens with COI≥15 were truly positive. In fact, when the COI value was between 0 and 0.99, 7 true positive samples were missed. According to the progress of HIV viremia and immune response after primary infection, Ag and Ab were detected at different times. Ab could not be detected in the 7 patients with acute HIV infection, therefore, COI values is less than 1.0.

For HIV1-p24 Ag, the positive COI value accounted for 21.56% of all screened samples, and the COI were mainly distributed in the range of 1-49.99 (98.01%). Nearly, COI was between 1 and 4.99, and 44.26% of the specimens were negative, while when the COI value was ≥5, more than 75% of the specimens were true positive. In particular, negative values are still possible at high levels of COI≥100.

## Discussion

This research is the first attempt to evaluate the clinical diagnostic performance of the Elecsys^®^ HIV Duo assay for HIV screening, to suggest the predictive value of COI for HIV infection and to confirm HIV infections in the context of a highly complex and multiethnic region of China. It is important to note that this study was carried out in southwest China, which has the highest number of HIV infection patients in the country, thus filling a gap in the lack of large-scale clinical evaluation of the Elecsys^®^ HIV Duo assay in southwest China.

From all the conclusive results in this research, a total of 186391 specimens were entered into our analysis, and 552 patients were confirmed to be positive for screening. The Elecsys^®^ HIV Duo assay, a 4th-generation assay, displayed 99.94%, 99.93% and 99.98% specificity of inpatient, outpatient and physical examination patients in detecting HIV patients from large and diverse clinical samples in China. Research subjects may be representative of a broad population, reflecting those who may present routinely testing in China. A study by Muehlbacher et al. (Muhlbacher et al., 2019) assessed the performance of the Elecsys^®^ HIV Duo assay at five international laboratories, and the specificity of blood donor samples was 99.87% and 100% in 1000 diagnostic samples. Similarly, a study (Zhang et al., 2020) also reported 99.93% specificity of the Elecsys^®^ HIV Duo assay. The high specificity of the Elecsys^®^ HIV Duo assay, as demonstrated, could thus identify potentially HIV-infected patients and guide the precise use of antiviral treatment early as possible for clinicians. Another study (Krasowski et al., 2021) also looked at the clinical performance of the Elecsys. The overall specificity of the Elecsys assay was 99.84% [95% CI 99.73-99.91 (8129/8142)]. Furthermore, the high specificity is a crucial characteristic of the Elecsys^®^ HIV Duo assay, which reduces the likelihood of false positives and not only meets the evaluation criteria set by the CDC but also reduces the number of additional Ab differential immunoassay confirmatory tests.

Previous studies (Kiely et al., 2010) showed that as the signal ratio of the negative control to the positive control, a high signal-to-cutoff (S/CO) ratio was predictive of confirmed HIV-positive results. Accordingly, this study also computed various overlap COIs between false positive and confirmed positive results, which suggests the predictive value of COI on HIV infection status. This study demonstrated that median COI ratios between true-positive and true-negative results of HIV Duo and HIV-1/2 Ab were large and false-positive group is significantly lower than that in the true-positive group. For HIV Duo, 99.49% (392/394) of individuals with COI ratios ≥50 were confirmed to be true-positive for HIV infection, and 97.85% of the specimens with COI ratios <15 were negative at the initial screening, verifying that high COI ratios were predictive of confirmed positive results. 15.0 were false positive results, suggesting that no additional laboratory tests are needed to confirm the results, especially in weakly responsive samples. Reasons for false positivity include small sample size, sample handling and variation in fixation methods, such as sample contamination and mislabeling. However, it is worth noting that although the COI ratio has predictive value for HIV infection, it is only an auxiliary method and cannot be used as a diagnostic tool. When doctors communicate with patients, they need to avoid increasing the psychological burden of patients and causing unnecessary medical disputes. If the patient had an epidemiological history, especially if the single Ag is positive, the patient should receive additional testing to determine HIV infection status. It has been reported that NAT detection can be directly applied to serum with COI level less than 4.0 (Parker et al., 2019).

According to the HIV screening recommendation, once the initial screening result is positive, the patient must be retested and confirmed to determine the final HIV infection status. Most patients with high COI levels (e.g., COI > 200.0) can be diagnosed directly by the WB confirmatory test.

3th-generation tests have been the primary means of screening for HIV infection in most parts of China. However, compared to other 4th- or 3rd-generation tests, the Elecsys^®^ HIV Duo assay has many advantages, such as excellent specificity, the ability to detect HIV-1 p24 Ag and HIV antibodies separately and a significantly shorter diagnostic window. Therefore, it can not only effectively and accurately diagnose early HIV infection but also distinguish between previously and newly reported infections. In the long term, widespread use of the method can help clinicians accurately identify and treat the disease, and early public health interventions can reduce the risk of transmission. In the area of diagnostic surveillance for the clinical management of HIV, the necessary cost savings can be achieved through the adoption of the Elecsys^®^ HIV Duo assay, which uses advanced, well-coordinated modern technology to greatly improve the quality of test results. Diagnostic algorithms with superior performance deserve more support to avoid wasting limited medical resources and funds on expensive and/or untested methods (Petti et al., 2006; Brown, 2007). At the same time, laboratory workers should pay attention to and participate in more reasonable algorithm design and method evaluation, rather than overly ambitious laboratory testing work. We hope that more scientific and reasonable detection algorithms and methods can be widely used in resource-limited areas.

There are several limitations to our study. First, this is a large retrospective study; 26 patients did not go through a complete algorithm, some patients may have been infected with HIV, and others may have been confirmed. No follow-up reasons may include loss of follow-up, death and unwillingness to accept diagnosis, which may affect our final results. Therefore, it is necessary to store and retrieve the results based on computers for the laboratory of a large number of specimens and the study of large data. Then, one of the limitations of our study is that there is no independent gold standard for determining “true negative”. A study conducted by Muhlbacher (Xu et al., 2020) et al. evaluated the performance of the Elecsys ^®^ HIV Duo assay in five international centres and compared with other available 4th-generation tests. The evaluation by the 139 seroconversion panels indicated that the Elecsys ^®^ HIV Duo test was highly sensitive. Therefore, we decided to just perform a specificity analysis. Finally, the original data come from a single hospital and are not fully representative or applicable to the entire local or even national population. We hope to expand the number and diversity of samples by collecting data in different regions.

## Conclusion

The Elecsys^®^ HIV Duo test (Cobase 801 analyzer) differentiates the detection of HIV Ag and anti-HIV antibodies with high specificity and facilitates the diagnosis of patients with early HIV infection. COI values are good predictors of HIV infection, with a high COI ratio predicting a confirmed positive result and 15.0 being a false positive result, indicating that no additional laboratory testing is required to confirm the result. Therefore, the Elecsys^®^HIV Duo test is an improvement of HIV Ag and Ab discrimination, making it suitable for routine clinical diagnosis.

## Data Availability Statement

The original contributions presented in the study are included in the article/supplementary material. Further inquiries can be directed to the corresponding author.

## Author Contributions

MY conceived and designed the study, and helped to draft and revise the manuscript for important intellectual content. CT and WY participated in study conception and design, collected data, performed statistical analysis and interpretation, and drafted and revised the manuscript. WS participated in study conception and design and helped to draft and revise the manuscript. MY prepared and collected data and helped to draft the manuscript. All authors made substantial contribution. All authors contributed to the article and approved the submitted version.

## Conflict of Interest

The authors declare that the research was conducted in the absence of any commercial or financial relationships that could be construed as a potential conflict of interest.

## Publisher’s Note

All claims expressed in this article are solely those of the authors and do not necessarily represent those of their affiliated organizations, or those of the publisher, the editors and the reviewers. Any product that may be evaluated in this article, or claim that may be made by its manufacturer, is not guaranteed or endorsed by the publisher.
